# PA-MSHA induces inflamed tumor microenvironment and sensitizes tumor to anti-PD-1 therapy

**DOI:** 10.1038/s41419-022-05368-6

**Published:** 2022-11-07

**Authors:** Min Huang, Fang He, Dan Li, Ya-Jia Xie, Ze-Bo Jiang, Ju-Min Huang, Xiao-Ping Zhao, Ali Adnan Nasim, Jun-Hui Chen, Jin-Cai Hou, Xian-Ming Fan, Elaine Lai-Han Leung, Xing-Xing Fan

**Affiliations:** 1 Dr. Neher’s Biophysics Laboratory for Innovative Drug Discovery, State Key Laboratory of Quality Research in Chinese Medicine, Macau University of Science and Technology, Macau, China; 2 Department of Respiratory and Critical Care Medicine, Affiliated Hospital of Southwest Medical University, Luzhou, Sichuan China; 3 Beijing Wante’er Biological Pharmaceutical Co.,Ltd., No.32 yard, East 2nd Road, Yanqi Economic Development Zone, Huairou District, Beijing, China; 4 Intervention and cell therapy center, Peking University ShenZhen Hospital, ShenZhen, Guangdong China; 5 Guangdong-Hong Kong-Macau Joint Lab on Chinese Medicine and Immune Disease Research, GuangZhou, Guangdong China

**Keywords:** Antigen processing and presentation, Cancer microenvironment

## Abstract

A low response rate to immune checkpoint inhibitor (ICI) therapy has impeded its clinical use. As reported previously, an inflamed tumor microenvironment (TME) was directly correlated with patients’ response to immune checkpoint blockade (ICB). Thus, restoring the cytotoxic effect of immune cells in the TME is a promising way to improve the efficacy of ICB and overcome primary resistance to immunotherapy. The effect of *Pseudomonas aeruginosa mannose-sensitive-hemagglutinin* (PA-MSHA) in facilitating T cell activation was determined in vitro and in vivo. Subsets of immune cells were analyzed by flow cytometry. Proteomics was carried out to comprehensively analyze the discriminated cellular kinases and transcription factors. The combinational efficacy of PA-MSHA and αPD-1 therapy was studied in vivo. In this study we demonstrated that PA-MSHA, which is a clinically used immune adjuvant, effectively induced the anti-tumor immune response and suppressed the growth of non-small cell lung cancer (NSCLC) cells. PA-MSHA showed great potential to sensitize refractory “cold” tumors to immunotherapy. It effectively enhanced macrophage M1 polarization and induced T cell activation. In vivo, in combination with αPD-1, PA-MSHA suppressed tumor growth and prolonged the survival time of allograft model mice. These results indicate that PA-MSHA is a potent agent to stimulate immune cells infiltration into the TME and consequently induces inflammation in tumors. The combination of PA-MSHA with αPD-1 is a potential strategy to enhance the clinical response rate to ICI therapy.

## Introduction

Employing immune checkpoint inhibitors (ICIs), like programmed cell death ligand 1 (PD-L1) and programmed cell death 1 (PD-1) antibodies, has drastically improved clinical outcomes for tumor patients [1, 2]. However, less than 30% of patients respond positively to ICIs and obtain encouraging results [3–5]. The immunosuppressive tumor microenvironment (TME) was reported as a major limitation for ICIs, which largely reduces the number and activation of effector T cells [6]. The lack of tumor-infiltrating lymphocyte (TIL) T cells result in so-called “cold” tumors, and remarkably contributes to tumor immune escape and compromise the clinical efficacy of ICIs. The causes of “cold” tumors include lack of tumor antigen, defect in antigen presentation, T cell exhaustion etc. [7]. Therefore, the ability to convert immune “cold” tumors into “hot” tumors, by increasing TILs, is urgently required to be studied.

*Pseudomonas aeruginosa mannose-sensitive-hemagglutinin* (PA-MSHA) is a gram-negative [8], *pseudomonas aeruginosa* bacterium genetically engineered with mannose-sensitive type 1 fimbriae on its surface [9, 10]. PA-MSHA injection has been approved in China as an immune adjuvant for treating patients with malignant tumors for many years [11, 12]. PA-MSHA is reported to possess anti-tumor and anti-inflammation effects. It has been reported that PA-MSHA could increase the ratio of M1/M2 in gastric and bladder cancer and strengthen the immune response against cancer [13, 14]. Macrophages are immune cells which are able to adopt different functional phenotypes in response to cytokine or pathogenic signals [15]. M2, also known as tumor-associated macrophages (TAMs), secrete molecules that inhibit anti-tumor immune response while promoting angiogenesis and tumor growth [16]. In contrast, classically activated M1 macrophages suppress tumor growth by releasing pro-inflammatory factors [17–19]. However, the mechanism by which PA-MSHA causes immune stimulation has not been completely elucidated, especially whether it can sensitize tumors to ICIs therapy.

In this study, we found that treatment of PA-MSHA for NSCLC has effectively reprogrammed “cold” TME into inflamed immune cell–infiltrated TME. PA-MSHA significantly increased anti-tumor immunity through polarization of macrophages toward the M1 phenotype by activating NF-κB /TLR5 pathway. Furthermore, the anti-tumor immune activities of PA-MSHA were examined in vitro and in vivo NSCLC model. Especially, combination with PA-MSHA significantly enhanced the anti-tumor effect of αPD-1 in mouse model.

## Materials and methods

### Reagents

Fetal bovine serum (FBS), RPMI 1640 medium, DMEM medium and antibiotics (Penicillin Streptomycin Glutamine solution) were purchased from GIBCO (Carlsbad, CA, USA). The human bronchial epithelial cell line BEAS-2B, KRAS-mutant human lung cell lines H358, H460 and A549, EGFR-mutant human lung cell lines PC-9, H1975, H1650 and THP-1 cell lines were purchased from American Type Culture Collection (ATCC, Manassas, VA, USA) and maintained at 37 °C with 5% CO_2_ in RPMI 1640 medium or DMEM medium supplemented with 10% FBS. PA-MSHA were provided by Beijing Wante’er Biological Pharmaceutical Co. Ltd. The anti-mouse PD-1 mAb was purchased from Bio X Cell (Lebanon, NH, USA). The PE/Cy7 anti-human CD14 antibody, FITC anti-human CD86 antibody, PE/Dazzle^TM^ 594 anti-human TNF-α antibody, PerCP anti-mouse CD45 Antibody, PE-Cy7 anti-mouse CD45 Antibody, APC anti-mouse CD3 Antibody, PE-CF594 anti-mouse CD4 Antibody, PE-CY7 anti-mouse CD8 Antibody, PE anti-T-bet Antibody, PE-CF594 anti-mouse TNF-α Antibody, PE-CF594 anti-mouse IFN-γ Antibody, FITC anti-mouse CD86 Antibody, PerCP anti-mouse CD11B Antibody, PE-CF594 anti-mouse MHC-II Antibody, PE-Cy5 anti-mouse F4/80 Antibody, PE anti-mouse CD11C Antibody, PE anti-mouse CD206 Antibody, APC anti-mouse Granzyme B Antibody, FITC anti-mouse CD44 Antibody, PE anti-mouse CD62L Antibody and APC-H7 AVI for flow cytometry and the protein transport inhibitor Brefeldin A were purchased from BioLegend (San Diego, CA, USA). The primary antibodies of β-actin, Erk1/2, p-Erk1/2, JNK, p-JNK, p38, p-p38, PI3K, p-PI3K, AKT, p-AKT, NF-κB, p- NF-κB, SRC, p-SRC, STAT1, p-STAT1 were purchased from Cell Signaling Technology (Danvers, MA, USA). The anti-rabbit secondary antibodies were purchased from Invitrogen (Carlsbad, CA, USA). The Transcription Universal cDNA Master kit and FastStart Universal SYBR Green Master (Rox) kit were purchased from Roche (Mannheim, Germany). The primers for TLR1-10, IL-1β, IL-6, IL-12α, IL-12β, CXCL9-11 and GAPDH were purchased from BGI (Beijing, China). The NF-κB inhibitor BAY 11-7082, TLR4 inhibitor Resatorvid, TLR5 inhibitor TH1020 and TLR8 inhibitor CU-CPT-8m were purchased from MedChem Express (Monmouth Junction, NJ, USA). Phorbol 12-myristate 13-acetate (PMA) and Lipopolysaccharides (LPS) were purchased from Sigma (ST. LOUIS, MO, USA).

### Cytotoxicity assay

3000 cells per well were seeded in a 96-well plate and incubated overnight to allow cells to adhere. Cells (BEAS-2B, A549, H358, H460, PC-9, H1975, H1650) were treated in different concentrations of PA-MSHA for 72 hours. 5 μL MTT (5 mg/ml) solution were added to each well for 4 hours. After that, 100 μL DMSO was added to each well for 10 minutes. Absorbance was measured at 490 nm in a microplate reader (Tecan, Morrisville, NC, USA).

### Co-culture of THP-1-derived macrophages and PA-MSHA

THP-1 cells (1 × 10^6^ cells/well) were seeded in 6-well plate and incubated with PMA for 24 h followed by resting in fresh medium for 24 h to induce M0. PA-MSHA were co-cultured with M0 macrophages for different periods of time for different detection targets as described below.

### Western blot

The cells were harvested and lysed in RIPA lysis buffer with protease inhibitors to extract total protein. The proteins were separated by 10% SDS-PAGE, followed by transferring to nitrocellulose membrane, and blocking with 5% skim milk for 1 hour. The primary antibodies were incubated overnight at 4°C. Then the anti-rabbit IgG HRP-linked secondary antibody were incubated for 1 hour at room temperature. The protein expression was detected by using the Amersham Imager 600 scanner (USA) after adding ultra-sensitive ECL chemiluminescent substrate (4 A BIOTECH, Beijing, CHINA).

### Q-RT-PCR

M0 macrophages were treated with PA-MSHA (1 × 10^8^ pcs/ml) for 6 hours, 12 hours, and 24 hours respectively. Then the total RNA was extracted by the Trizol (Invitrogen, USA). Transcription Universal cDNA Master kit (Roche) was used to obtain cDNA by reverse transcription according to the instructions, and q-PCR was performed with FastStart Universal SYBR Green Master kit (Roche) according to the instructions. The sequences of the primers for TLR1-10, IL-1β, IL-6, IL-12α, IL-12β, CXCL9-11 and GAPDH were shown in Table 1.

**Table 1:** Primer sequences of TLR1-10, IL-1β, IL-6, IL-12α, IL-12β, CXCL9-11 and GAPDH.

TLR1	5’-CAGCGATGTGTTCGGTTTTCCG-3’
	3’-GATGGGCAAAGCATGTGGACCA-5’
TLR2	5’-CTTCACTCAGGAGCAGCAAGCA-3’
	3’-ACACCAGTGCTGTCCTGTGACA-5’
TLR3	5’-GCGCTAAAAAGTGAAGAACTGGAT-3’
	3’-GCTGGACATTGTTCAGAAAGAGG-5’
TLR4	5’-CCCTGAGGCATTTAGGCAGCTA-3’
	3’-AGGTAGAGAGGTGGCTTAGGCT-5’
TLR5	5’-CCTTACAGCGAACCTCATCCAC-3’
	3’-TCCACTACAGGAGGAGAAGCGA-5’
TLR6	5’-GTCCAGAGTGAGTGGTGCCATTAC-3’
	3’-GCCTTCAGCTTGTGGTACTTGTTG-5’
TLR7	5’-CACAGCCGTCCCTACTGTTT-3’
	3’-TTTTTACACGGCGCACAAGG-5’
TLR8	5’-ACTCCAGCAGTTTCCTCGTCTC-3’
	3’-AAAGCCAGAGGGTAGGTGGGAA-5’
TLR9	5’-TGAGCCACAACTGCATCTCGCA-3’
	3’-CAGTCGTGGTAGCTCCGTGAAT-5’
TLR10	5’-GGTTAAAAGACGTTCATCTCCACG-3’
	3’-CCTAGCATCCTGAGATACCAGG-5’
IL-1β	5’-CCACAGACCTTCCAGGAGAATG-3’
	3’-GTGCAGTTCAGTGATCGTACAGG-5’
IL-6	5’-AGACAGCCACTCACCTCTTCAG-3’
	3’-TTCTGCCAGTGCCTCTTTGCTG-5’
IL-12α	5’-TGCCTTCACCACTCCCAAAACC-3’
	3’-CAATCTCTTCAGAAGTGCAAGGG-5’
IL-12β	5’-GACATTCTGCGTTCAGGTCCAG-3’
	3’-CATTTTTGCGGCAGATGACCGTG-5’
CXCL9	5’-CTGTTCCTGCATCAGCACCAAC-3’
	3’-TGAACTCCATTCTTCAGTGTAGCA-5’
CXCL10	5’-GGTGAGAAGAGATGTCTGAATCC-3’
	3’-GTCCATCCTTGGAAGCACTGCA-5’
CXCL11	5’-AAGGACAACGATGCCTAAATCCC-3’
	3’-CAGATGCCCTTTTCCAGGACTTC-5’
GAPDH	5’-GTCTCCTCTGACTTCAACAGCG-3’
	3’-ACCACCCTGTTGCTGTAGCCAA-5’

### Agarose gel electrophoresis of RT-qPCR products

5 μL RT-qPCR products were mixed with 5 μL 2 × loading dye. 10 μL mixture were loaded and separated on 1% agarose gel by electrophoresis and visualized under UV-light.

### TLRs and NF-κB blockade

To block the NF-κB pathway and TLRs in macrophages, the NF-κB inhibitor BAY 11-7082 or TLRs inhibitors (Resatorvid for TLR4, TH1020 for TLR5, CU-CPT-8m for TLR8) were added 1 hour before PA-MSHA treatment. TNF-α and IL-6 levels in macrophages were detected by flow cytometry and q-PCR respectively.

### Flow Cytometry analysis

To detect the M1 macrophage ratio, the THP-1 cells were resuspended and stained with PE/Cy7 anti-human CD14 antibody and FITC anti-human CD86 antibody for 30 minutes after PA-MSHA treatment. To determine the expression of TNF-α, the THP-1 cells were treated with protein transport inhibitor Brefeldin A (5 μg/ml) for 4 hours before the terminal of PA-MSHA treatment, then collected the cells, fixed with 1× true nuclear fixation buffer (BioLegend, CA) for 1 hour and stained with PE/DazzleTM594 anti-human TNF-α antibody diluted in 1× true nuclear permeabilization buffer (BioLegend, CA) for 30 minutes. All samples were analyzed by BD FACS Aria III flow cytometry.

For FACS analysis, single-cell suspensions were stained with the following antibodies: stained with Fix/Viable eF780 and blocked with the CD16/CD32 antibody, and then stained with the CD45 Antibody, CD3 Antibody, CD4 Antibody, CD8 Antibody, CD44 Antibody, CD62L Antibody, F4/80 Antibody, CD11b Antibody, CD86 Antibody, CD206 Antibody, CD14 Antibody at 4 °C for 30 min. TNF-α Antibody, IFN-γ Antibody, Granzyme B Antibody, MHC-II Antibody, HLA-DR Antibody, T-bet Antibody staining were performed using the Intracellular Fixation and Permeabilization kit according to the manufacturer’s instructions. After being washed, all samples were resuspended in phosphate-buffered saline (PBS) and detected by BD FACS Aria III flow cytometry.

### Proteomic analysis

The total proteins of THP-1-derived macrophages treated with or without PA-MSHA were extracted with RIPA lysis buffer containing protease inhibitors, followed by precipitated with methanol and chloroform. Proteins (100 µg) were reduced and alkylated with 1 mM dithiotreitol and 0.5 mM iodoacetamide, respectively, then trypsin digested overnight in the ratio of 1:100. The peptides were desalted, dry in vacuum and dissolved for LC-MS/MS analysis The MS data were analyzed with MaxQuant software (version 1.5.5.1).

### Animal studies

All animal studies adopt animal ethics and use experimental animals. For different dose of PA-MSHA experiment, approximately 5 × 10^5^ Luciferase Lewis lung cancer (LLC) cells were injected subcutaneously into 6–8 weeks of male C57bl/6 J mice. A total of 24 mice were randomized to receive one of the following treatments by intraperitoneal injection (every five days injection when tumors reached a volume of 20mm^3^, total for five times): control group (PBS), PA-MSHA group (2 × 10^8^ pcs/ml), PA-MSHA group (4 × 10^8^ pcs/ml), PA-MSHA group (8 × 10^8^ pcs/ml). For PA-MSHA combined αPD-1 experiment, approximately 5 × 10^5^ Luciferase LLC cells were injected subcutaneously into 6–8 weeks of male C57bl/6 J mice. A total of 24 mice were equally divided into four groups: control group (PBS), αPD-1 (250 μg), PA-MSHA (4 × 10^8^ pcs/ml) and αPD-1(250 μg) plus PA-MSHA (4 × 10^8^ pcs/ml). Every five days injection of PA-MSHA and treatment with αPD-1 when tumors reached a volume of 20mm^3^. Tumor growth was monitored every 3 days by measuring tumor length (L) and width (W). Tumor volume (V) was then calculated using the formula, V = 1/2 × L × W^2^. Tumors and blood were collected for pharmacodynamic analysis on day 21. We analyzed the number of T cells and macrophage cells in combination-treated mice. Moreover, to analyze survival, we treated mice with drugs (as above) for 7 to 10 weeks. The survival endpoint of tumor-bearing mice was reached when the primary tumor volume was greater than 2000 mm^3^, or when the animal demonstrated signs of severe pain and discomfort, or when the animal died because of disease progression.

### In vivo Xtreme imaging

Xtreme imaging was performed after treatment. The mice were injected 150 μL luciferin and after 5 min 100 μL 1% pentobarbital sodium was injected. After injection, three mice at a time were placed in the mouse bed of the imaging station for simultaneous imaging.

### Hematoxylin and Eosin (H&E) staining

5μm thick paraffin sections were stained with H&E according to the standard procedure. Tissue sections of tumors were observed under a Leica microscope. Leica camera (dfc310 FX) was used to capture images.

### T cell activation

The mice were sacrificed by cervical dislocation and the spleens were removed aseptically. The spleen cells were obtained by pushing PBS through the spleen with a syringe. All harvested cells were dissolved twice in erythron lysis buffer until most of the erythrocytes were removed. In T cell activation test, the freshly isolated primary splenocytes (2 × 10^6^/well) were cultured in complete medium in 24 well round plate, setting containing different concentrations PA-MSHA (1 × 10^6^ pcs/ml; 1 × 10^7^ pcs/ml; 1 × 10^8^ pcs/ml) and anti-CD3 (1 μg/ml)/anti-CD28 (5 μg/ml) for positive control. The cells were cultured for 72 hours at 37 °C in 5% CO_2_ and viewed under Leica microscope. Cells were harvested and stained for flow cytometry analysis.

### Statistical analysis

The data of three independent experiments were expressed as means ± standard deviation. Graph pad prism 8 used one-way analysis of variance (ANOVA) to determine the differences between groups, and then used Bonferroni test to compare all column pairs. The survival rate of Lewis lung cancer model mice was calculated by Kaplan Meier method, and the statistical significance found by log rank test. Results of *P* < 0.05 were considered statistically significant.

## Results

### PA-MSHA induced M1 macrophages polarization and enhanced T cell activity

**Fig. 1:**
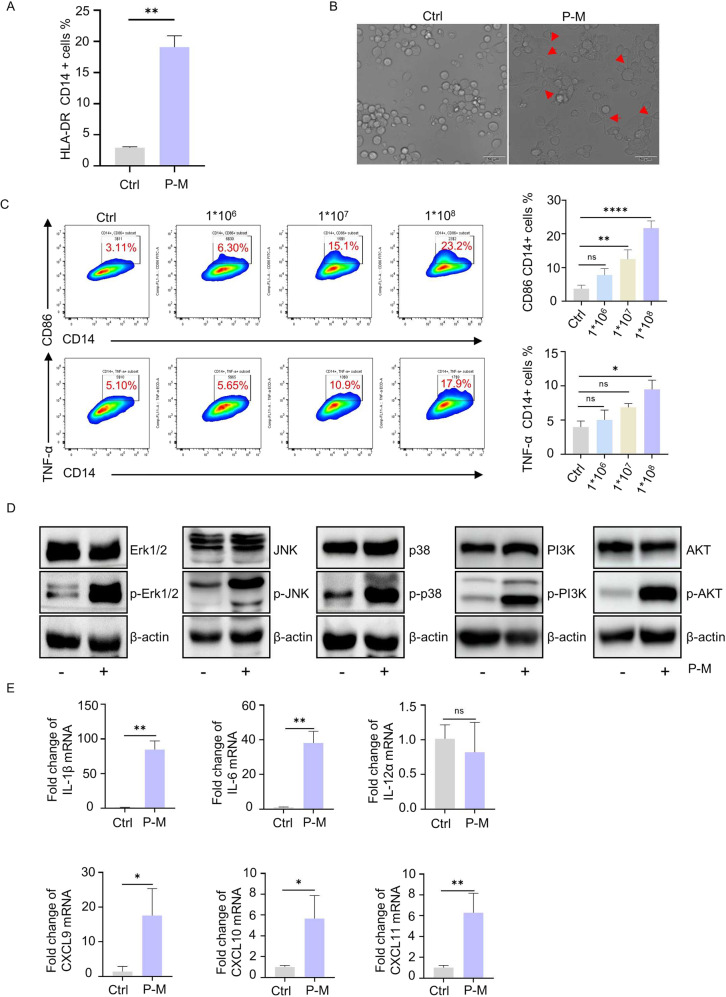
PA-MSHA induced M1 macrophage polarization. **A** The expression of HLA-DR on THP-1 cells were enhanced by PA-MSHA (1 × 10^8^ pcs/ml). **B** Cell morphology of THP-1 after co-culture with PA-MSHA was changed, pseudopodia growth marked by red arrows. **C** PA-MSHA upregulated M1 marker CD86 and TNF-α in THP-1-derived macrophages. **D** PA-MSHA induced the phosphorylation of PI3K/AKT and p38/JNK/Erk in THP-1 cells. **E** The mRNA level of IL-1β, IL-6, IL-12α and CXCL9-11 by q-PCR. P-M is short for PA-MSHA. (*P < 0.05, **P < 0.01, ***P < 0.001, ****P < 0.0001.).

PA-MSHA is a bacterium-derived immune adjuvant and servers as an exogeneous antigen for host immunity [20, 21]. Therefore, we hypothesized that it may stimulate antigen-presenting cells (APCs) and thus activate an anti-tumor immune response. We first investigated the expression of MHC-II, which is a representative marker for pro-inflammatory M1 polarization on macrophages and presents extracellular antigens for T cells. As shown in Fig. 1A, B, PA-MSHA significantly enhanced the level of HLA-DR, MHC-II cell surface receptors, and pseudopodia growth on THP-1 cells. This indicates the activation of macrophages. The levels of pro-inflammatory M1 marker CD86 and tumor-killing cytokine TNF-α were significantly up-regulated by PA-MSHA as well (Fig. 1C and Fig. S1A-B). PI3K/AKT and MAPK signaling, which are reported as critical kinases for inducing macrophage M1 polarization [22, 23], were significantly activated by PA-MSHA (Fig. 1D). In addition, cytokines IL-1β, IL-6, IL-12, and chemokines CXCL9-11, which suggesting the phenotype of inflammatory macrophages and the ability of activating lymphocytes [24, 25] also increased significantly in PA-MSHA treated group (Fig. 1E and Fig. S1C), further demonstrating the M1 polarization effects of PA-MSHA.

**Fig. 2:**
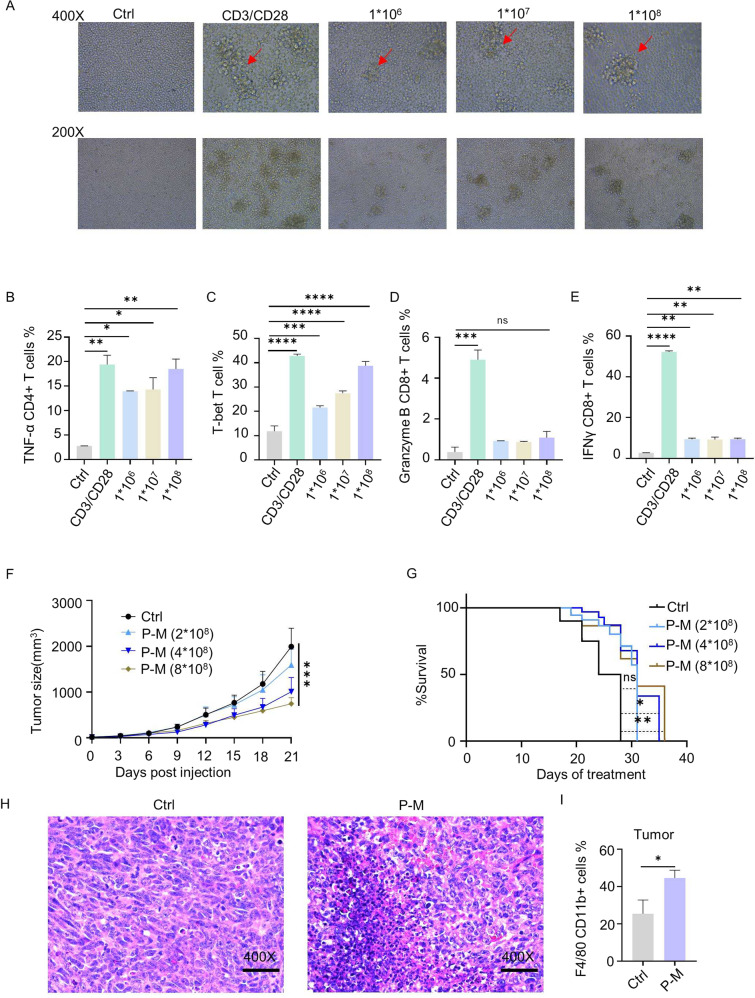
PA-MSHA increased tumor infiltrating immune effector cells and inhibits tumor growth in vivo. **A** T cells were activated by PA-MSHA at 72 hours, original magnification, ×400 and ×200. **B** TNF-α of CD4^+^ T cells (**C**) T-bet in T cells (**D**) Granzyme B in CD8^+^ T cells **E** IFN-γ levels in CD8^+^ T cells were all significantly increased by PA-MSHA. **F** In vivo assay, PA-MSHA inhibited tumor growth in a dose dependent manner. **G** PA-MSHA prolonged the survival time of tumor-bearing mice. **H** H&E analysis showed that PA-MSHA (4 × 10^8^ pcs/ml) induced more immune cells infiltration in tumor tissue. Original magnification, ×400. (**I**) Flow-cytometry data showed that the number of macrophages in LLC tumors was significantly upregulated by PA-MSHA. P-M is short for PA-MSHA. (*n* = 6 mice/group, *P < 0.05, **P < 0.01, ***P < 0.001, ****P < 0.0001.).

Subsequent CD4^+^ and CD8^+^ T cell activation level was determined by e*x vivo* assay. Immune cells isolated from the spleens of C57bl/6 J mice were cultured with different concentrations of PA-MSHA with anti-CD3/CD28Abs was used as a positive control. PA-MSHA remarkably stimulated aggregation and proliferation of immune cells (Fig. 2A). The gating strategies used in flow cytometry was presented in Fig. S2. The expression of TNF-α in CD4^+^ T cells (Fig. 2B), T-bet in T cells (Fig. 2C), as well as Granzyme B and IFN-γ in CD8^+^ T cells was remarkably increased by PA-MSHA (Figs. 2D and 2E). These results suggested that PA-MSHA is an effective agent to stimulate host immune response for anti-tumor therapy.

### PA-MSHA increased tumor-infiltrating immune effector cells and inhibited tumor growth in vivo

Inducing inflammation of tumors is one of the ways with the most potential to enhance the efficacy of ICIs [26]. Since PA-MSHA effectively induced pro-inflammatory status of immune cells, we examined whether PA-MSHA could recruit more immune cells to the TME and thus cause greater tumor suppression in lung tumor model mice.

Firstly, the safety of PA-MSHA was assessed on numerous types of NSCLC cells and normal cells. No significantly cytotoxic effect on both tumor and normal cells was observed even at the high dose of 1 × 10^8^ pcs/ml (Fig. S3A). It was supported by in vivo results which showed that body weights of PA-MSHA-treated mice did not differ significantly from those of the control group (Fig. S3B). The efficacy of PA-MSHA was determined as well. Treatment with PA-MSHA remarkably suppressed growth of tumors in vivo and prolonged the survival time of the tumor-bearing mice (Fig. 2F, G). H&E staining for tumor tissue was compared between control and PA-MSHA treated group. Interestingly, there were significantly more immune cells in PA-MSHA treated samples than in the control group, indicating more immune cells were recruited to the TME (Fig. 2H). The results were further supported by flow cytometry analysis which showed that the number of macrophages from TME was increased nearly 1.5-fold (Fig. 2I and Fig. S3C). These suggest that PA-MSHA increased the infiltration of immune cells into the TME and potentially inflamed tumors.

### PA-MSHA induced M1 macrophage polarization by targeting TLR5 and activating the NF-κB signaling pathway

**Fig. 3:**
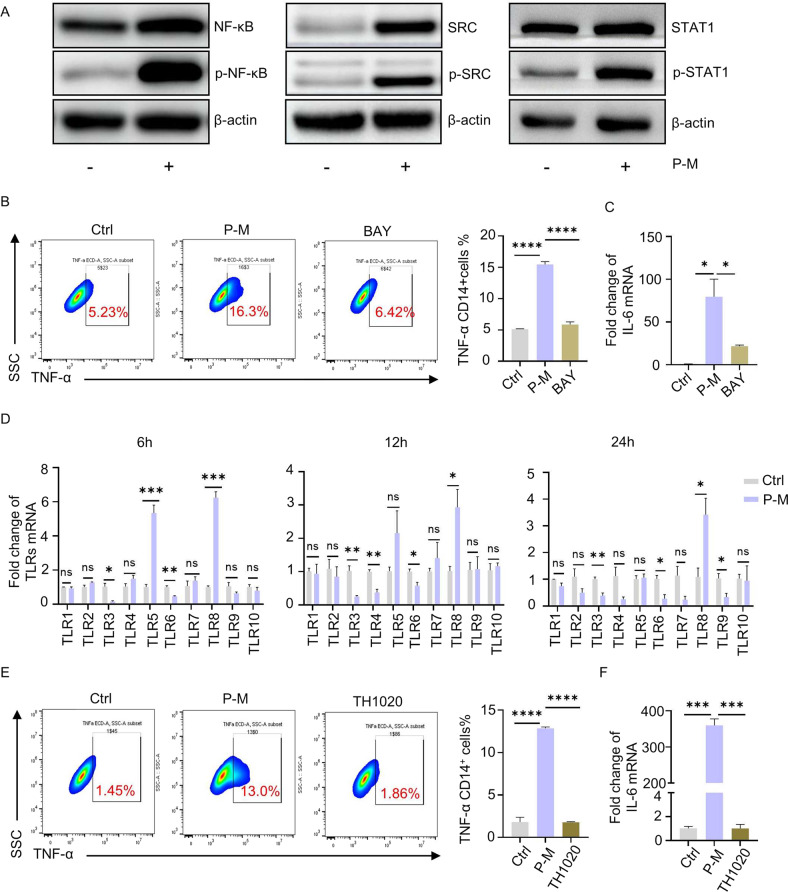
PA-MSHA induced M1 macrophage polarization by targeting TLR5 and activating the NF-κB signaling pathway. **A** Confirmation of NF-κB, SRC, STAT1 changes by Western-Blot. **B**–**C** NF-κB blockade in THP-1 cells suppressed expression of TNF-α and IL-6. **D** mRNA change of TLRs in THP-1 derived macrophages after treatment with PA-MSHA for 6 hours, 12 hours and 24 hours. **E**–**F** Inhibitor of TLR5 (TH1020, 5 μM) suppressed the expression of TNF-α and IL-6. P-M is short for PA-MSHA. (*P < 0.05, **P < 0.01, ***P < 0.001, ****P < 0.0001.).

To explore the molecular mechanism of induction of M1 polarization by PA-MSHA, proteomics analysis was applied to determine the differentially expressed proteins. As shown in Table 2, various proteins were up-regulated in PA-MSHA-treated THP-1 cells. By clustering analysis of these differential proteins, NF-κB, STAT1, and SRC were identified as three mostly correlated targets (Fig. S4). Immuno-blotting results also confirmed the regulating effect of PA-MSHA on these targets (Fig. 3A). We further investigated the function of these three proteins in M1 polarization of macrophages. Pharmacological inhibitors of NF-κB (BAY 11-7082), STAT1 (Fludarabine) and SRC (BMS-354825) were used to block the effect of PA-MSHA. The results showed that the expression of its downstream targets, TNF-α and IL-6 which were significantly upregulated by PA-MSHA, was suppressed by BAY 11-7082 (Figs. 3B, 3C and S5A). This suggests the critical role of NF-κB in PA-MSHA-induced M1 differentiation.

**Table 2:** Proteins Significantly up regulated in PA-MSHA treated THP-1cells by proteomics analysis.

Signaling pathway description	*P value*	Count	Name of proteins
hydrolase activity, acting on carbon-nitrogen (but not peptide) bonds, in cyclic amidines	1.73E-06	5	AMPD2; MTHFD2; MTHFD2L; AMPD3; AMPD1
non-membrane spanning protein tyrosine kinase activity	8.05E-06	5	SRC; YES1; SRMS; FRK; PRKCD
protein tyrosine kinase activity	1.15E-05	8	SRC; YES1; SRMS; FRK; MET; MST1R; PRKCD; NRP1
cell adhesion molecule binding	3.53E-05	12	PXN; VAPB; IL1B; SRC; RPL34; ZC3HAV1; ATXN2L; FMNL2; STAT1; ANXA7; EHD1; ICAM1
protein kinase C binding	0.000212	4	SQSTM1; SRC; MARCKS; PLEK
hydrolase activity, acting on carbon-nitrogen (but not peptide) bonds	0.000261	6	CAD; AMPD2; MTHFD2; MTHFD2L; AMPD3; AMPD1
nuclear localization sequence binding	0.000556	3	KPNA2; IPO5; RANBP6
cadherin binding	0.00074	8	VAPB; SRC; RPL34; ZC3HAV1; ATXN2L; FMNL2; STAT1; EHD1
deaminase activity	0.00084	3	AMPD2; AMPD3; AMPD1
integrin binding	0.00098	5	PXN; IL1B; SRC; ANXA7; ICAM1
double-stranded RNA binding	0.001081	4	OAS2; EIF4B; RFTN1; OAS3
transferase activity, transferring pentosyl groups	0.002495	3	UMPS; NAMPT; UPP1
signal sequence binding	0.003001	3	KPNA2; IPO5; RANBP6
transferase activity, transferring glycosyl groups	0.006017	6	UMPS; GYS1; GALNT2; GALNT4; NAMPT; UPP1
transmembrane receptor protein tyrosine kinase activity	0.006745	3	MET; MST1R; NRP1
ubiquitin-like protein ligase binding	0.009672	6	PXN; SQSTM1; HGS; SRC; TRAF1; STAT1
transmembrane receptor protein kinase activity	0.012837	3	MET; MST1R; NRP1
cytokine receptor binding	0.027356	5	DDT; CXCL8; IL1B; TRAF1; STAT1
peptide binding	0.028835	5	CD74; KPNA2; TAP1; IPO5; RANBP6
cytokine activity	0.043182	4	CXCL8; TIMP1; IL1B; NAMPT
growth factor receptor binding	0.045337	3	IL1B; SRC; YES1
protein phosphatase binding	0.046187	3	PXN; MET; STAT1

Next, as toll like receptors (TLRs) are known to initiate the activation of innate and adaptive immunity [27], and NF-κB acts as its key downstream transcription factor [28], we verified which subtype of TLR responds to PA-MSHA and thus triggers NF-κB. As shown in Fig. 3D, PA-MSHA upregulated the expression of TLR5 and TLR8 among ten subtypes of TLRs in human macrophages. To demonstrate the critical role of TLRs in M1 polarization by PA-MSHA, inhibitors of TLR5 and TLR8 were used to block the effect of PA-MSHA. Interestingly, as the main cytokines of M1 macrophage, TNF-α and IL-6 were suppressed by TLR5 inhibitor (Figs. 3E and 3F). However, TLR8 inhibition (Fig. S5B-D) failed to counteract the effect of PA-MSHA. Moreover, inhibitor of TLR4, which is reported to participate in NF-κB activation during inflammation, also failed to block the effect of PA-MSHA (Fig. S5E-G). Therefore, based on the proteomics analysis and target blocking results, we concluded that PA-MSHA is a M1 macrophage activator by activating TLR5/NF-κB pathway.

### PA-MSHA boosted the efficacy of αPD-1 therapy in vivo

**Fig. 4:**
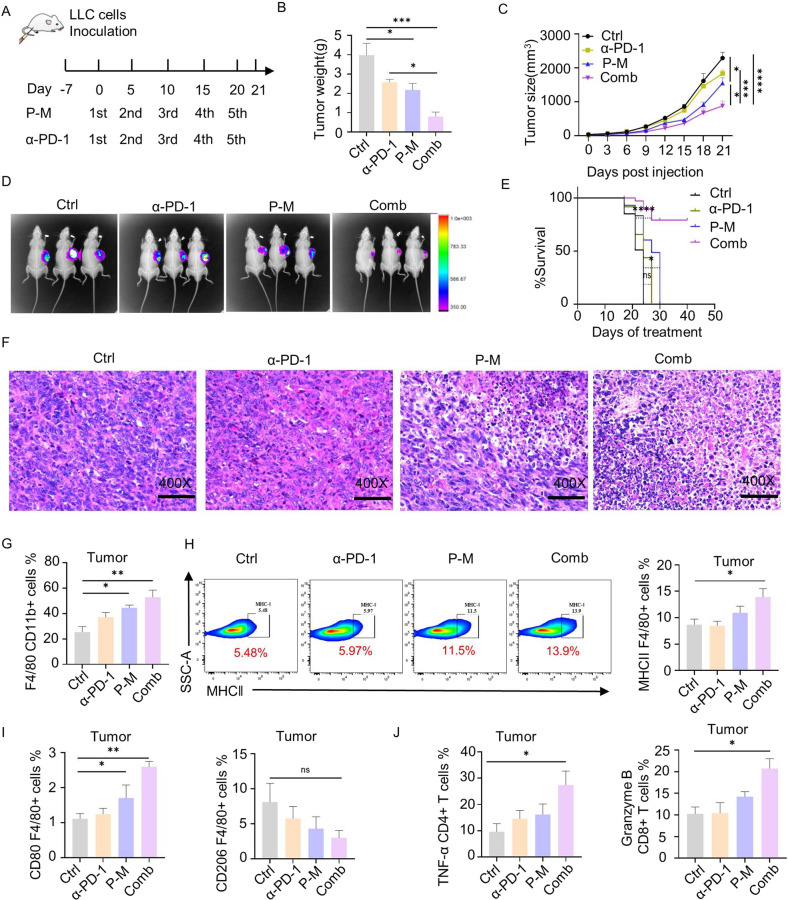
PA-MSHA boosted the efficacy of αPD-1 therapy in tumor. **A** Experimental strategy and schedule. Mice were subdivided into four groups: Control (injected with PBS), αPD-1, PA-MSHA, and PA-MSHA plus αPD-1. Treatment administered every five days injection from day 0 to day 21. Tumor tissues and PBMC were obtained on day 21. **B** Tumor weight and (**C**) Tumor size was remarkably inhibited in combinational group. **D** In vivo fluorescence images of LLC tumor-bearing mice at 21 days showed that the growth of tumor was significantly inhibited by combinational treatment. **E** Survival curve showing different groups of tumor-bearing mice. **F** H&E analysis of tumor tissue indicated that more immune cells were recruited into TME by PA-MSHA. Original magnification, ×400. **G** The number of macrophages and (**H**) their MHC-II level in LLC tumors were significantly enhanced by PA-MSHA and combinational treatment. **I** The number and activity of M1 macrophages, which represented by CD80 was remarkably increased in LLC tumors, while CD206, marker of M2 macrophages was downregulated by treatments. **J** The expression of TNF-α of CD4^+^ T cells and Granzyme B of CD8^+^T cells in different treatment groups. P-M is short for PA-MSHA. (*n* = 6 mice/group, *P < 0.05, **P < 0.01, ***P < 0.001, ****P < 0.0001.).

Since PA-MSHA effectively activates the immune system, we further investigated whether it contributed to αPD-1 therapy in vivo. PA-MSHA and αPD-1 were, alone or in combination, administered to a lung cancer allograft mouse model (Fig. 4A). The combined group achieved the most effective results. Tumor weight, tumor size, and intensity of tumor luciferase were remarkably decreased, while survival time (Fig. 4B–E) was significantly prolonged in the combined group.

Next, the stimulatory effect of immune cells in the TME was detected. H&E staining showed that more immune cells infiltrated into the TME in PA-MSHA or combined group (Fig. 4F). The infiltration of CD4 and CD8 T cells in tumors were significantly increased after combined administration (Fig. S6A-D). Compared to the single treatment group, the number of macrophages in the TME was increased by nearly two-fold, and MHC-II expressing cells were increased significantly indicating the expansion and activation of APCs (Fig. 4G-H). Interestingly, the percentage of macrophage subtypes was changed. The level of pro-inflammatory M1 macrophage was remarkably increased, whereas immune-suppressive M2 macrophages were decreased in combined treatment mice (Fig. 4I, Fig. S6E). For T cells, the anti-tumor activity of T cells in the TME was significantly enhanced. TNF-α in CD4^+^ T cells, GranzymeB in CD8^+^ T cells and IFN-γ in CD8^+^ T cells were up-regulated (Fig. 4J, Fig. S6F).

**Fig. 5:**
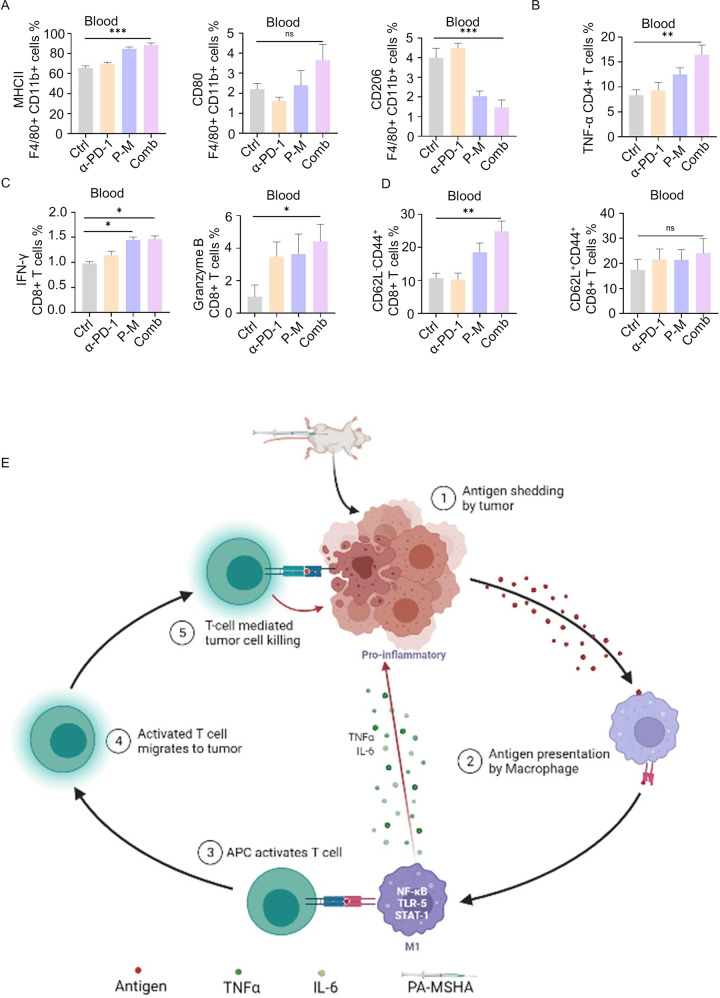
PA-MSHA enhanced the anti-tumor activity of PBMC. **A** The expression of MHC-II and CD80 macrophages was upregulated and CD206 macrophages was downregulated in combined group. **B** TNF-α of CD4^+^ T cells, **C** IFN-γ and Granzyme B of CD8^+^ T cells in blood were all up-regulated in blood tissues after different treatments. **D** Flow cytometry data showed an increase in effector memory T cells (CD62L^-^CD44^+^) and central memory (CD62L^+^CD44^+^) T cells specific to the CD8^+^ subset. **E** An overview schematic diagram showed how PA-MSHA modifies the tumor microenvironment and potentiates the antitumor effect. P-M is short for PA-MSHA. (*n* = 6 mice/group, *P < 0.05, **P < 0.01, ***P < 0.001, ****P < 0.0001.).

The changes in immune cells from PBMC were determined as well, the results of which corresponded with that of the TME. For antigen presenting cells, the level of MHC-II and number of M1 macrophages were remarkably enhanced, while immune-suppressing M2 macrophages decreased (Fig. 5A). The activity of both CD4 and CD8 T cells were remarkably up-regulated (Figs. 5B and 5C).

At last, we detected whether PA-MSHA can induce the proliferation of memory effect T cells, which contribute to preventing the relapse of tumors. As shown in Fig. 5D, the number of effector memory CD8^+^ T cells (CD62L^-^CD44^+^) and central memory CD8^+^ T cells (CD62L^+^CD44^+^) were significantly enhanced. These suggest that the combined treatment successfully established immune memory for tumor antigens. Collectively, we report that PA-MSHA and αPD-1 combination therapy can effectively induce inflammation in the TME by increasing the number of M1 macrophages and activating cytotoxic T cells, thus improving the anti-tumor efficacy of immunotherapy (Fig. 5E).

## Discussion

Non-inflamed subsets of tumors result in low responsiveness to current ICI immunotherapy and limit patients’ benefits from it. To maximize the efficacy of ICI immunotherapy, strategies for transforming a non-inflamed TME into a T cell inflamed TME are required. In this study, we demonstrate that PA-MSHA effectively converted immune “cold” (poorly infiltrated) tumors into inflamed (highly infiltrated) ones. As an immune adjuvant, PA-MSHA increases immune recognition of tumor cells [29], macrophage M1 polarization and T cell activation in vitro and in vivo. It enhances anti-PD-1 efficacy by increasing the number of TME infiltrating effector T cells and the response rate of immunotherapy, which significantly improved anti-tumoral responses and prolonged animal survival. Promisingly, PA-MSHA did not show significant cytotoxic effects for normal cells even at the high dose of 1 × 10^8^ pcs/ml. Since PA-MSHA is already a CFDA-approved agent, this is a promising strategy to enhance the clinical efficacy of ICI therapy.

The mechanism by which PA-MSHA functions is by specifically triggering the TLR5/NF-κB signal pathway in antigen-presenting cells and consequently stimulating an immune response to a tumor. The TLR5/NF-κB signal promotes M1 macrophage transformation, while reducing immunosuppressive M2 macrophage levels. Macrophages are the main immunomodulatory cells, which directly interact with T cells in the TME [15]. M1 macrophages promote T cell activity and infiltration in TME: there was a significant increase in the number of cytotoxic T cells (Granzyme B /IFN-γ CD8^+^) infiltrating tumors of mice treated with PA-MSHA and αPD-1 when compared with the control.

TLRs trigger the activation of innate immunity by recognizing endogenous ligands from pathogens. Many subsets of TLR have been reported to be closely related with tumor progression and thus specific agonists have been developed for tumor immunotherapy [30, 31]. For example, downregulation of TLR5 in triple-negative breast cancer (TNBC) increased tumor invasiveness and epithelial–mesenchymal transition (EMT) expression and promoted TNBC metastasis [32, 33]; TLR9 agonist CpG oligonucleotide was widely shown to have an anti-tumor effect by stimulating local immunity [34, 35]. Therefore, TLRs are potential target for tumor immunotherapy. TLR5 is known to recognize flagellin of invading bacteria and activate immune response [36]. PA-MSHA is a genetically engineered bacterium with mannose-sensitive type 1 fimbriae which differ from *pseudomonas aeruginosa* and confer its anti-tumor ability [37]. Further study is required to identify which peptide of bacterial flagellin in PA-MSHA is responsible for promoting TLR5 expression. Down-stream of TLR the phosphorylation of NF-κB was remarkably up-regulated. Interestingly, when NF-κB was blocked with specific inhibitor, the levels of TNF-α, IL-6 and other cytokines were down-regulated. This indicates that the activation of NF-κB plays a key role in the polarization of M1 macrophages by PA-MSHA.

Taken together, this new combinational therapy could be a promising alternative approach for improving therapeutic effects, especially for those patients with NSCLC who were unsatisfied with immune-modulatory therapy. Our study provides a leading-edge approach to combining bacterial drugs with immune checkpoint blockade, which will contribute to cancer immunotherapy for patients.

## Supplementary information

original WB figure3A

original WB figure1D

supplementary legends

original WB supplementary figure 5A

Authors’ contributions

checklist

Supplementary Figure 1

Supplementary Figure 2

Supplementary Figure 3

Supplementary Figure 4

Supplementary Figure 5

Supplementary Figure 6

## Data Availability

The datasets used and/or analyzed during the current study are available from the corresponding author on reasonable request.
