# Supplementary figures and images for: PA-MSHA induces inflamed tumor microenvironment and sensitizes tumor to anti-PD-1 therapy
Source: Cell Death Dis. 2022 Nov 7;13(11):931. doi: 10.1038/s41419-022-05368-6 (PMC9640707; doi:10.1038/s41419-022-05368-6)

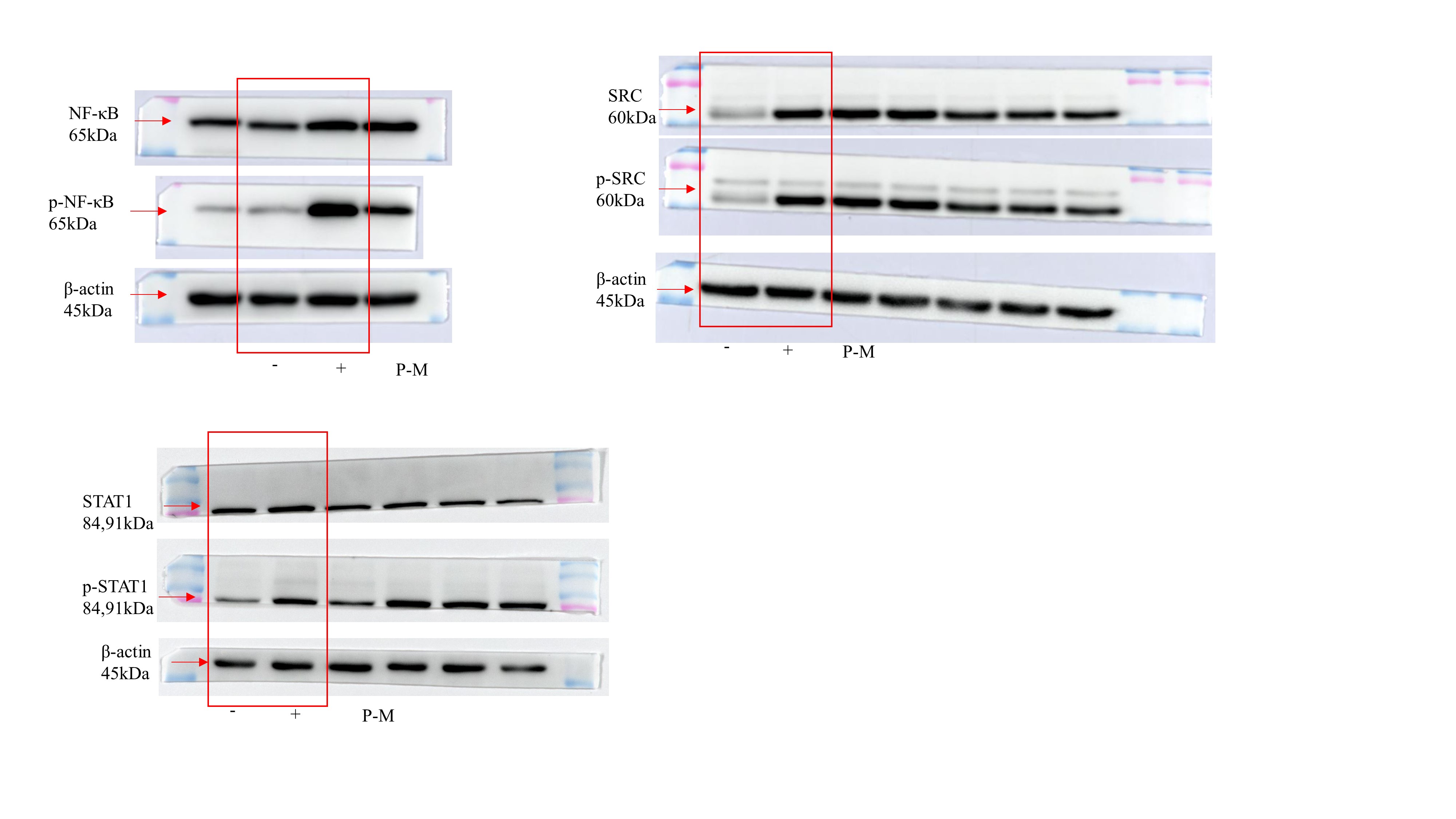

Supplement: Supplementary file 1 — original WB figure3A [file 41419_2022_5368_MOESM1_ESM.jpg]

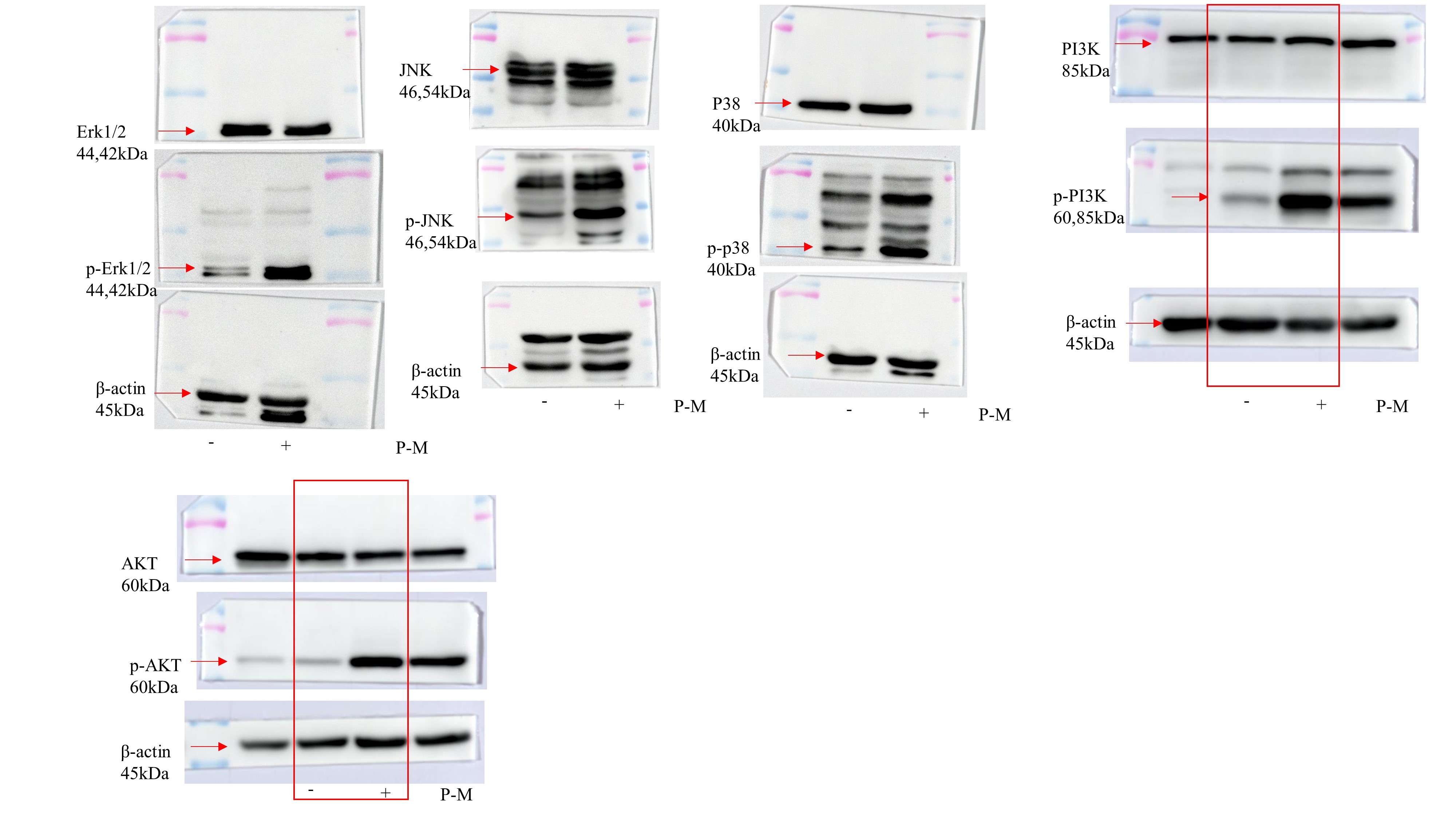

Supplement: Supplementary file 2 — original WB figure1D [file 41419_2022_5368_MOESM2_ESM.jpg]

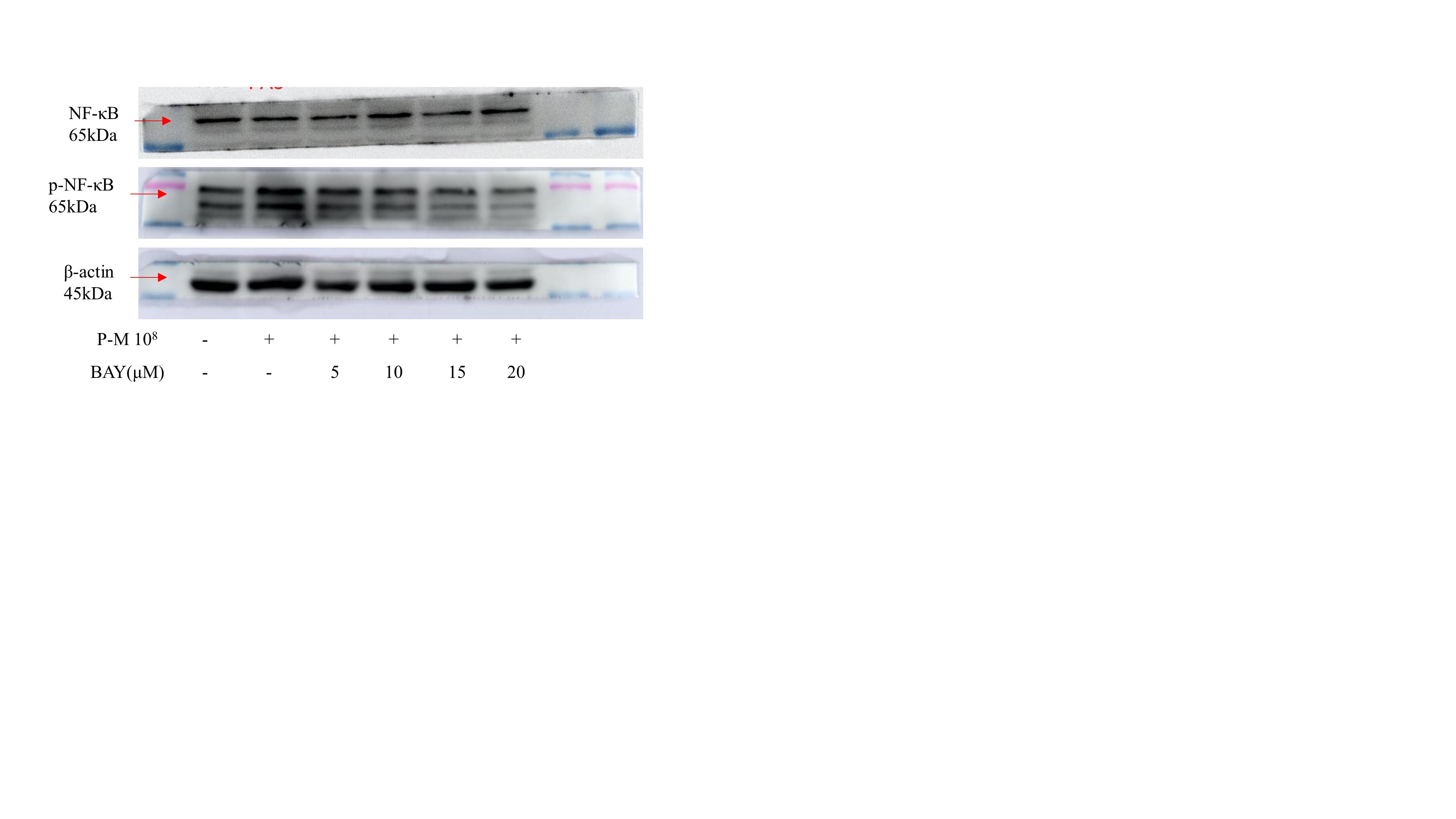

Supplement: Supplementary file 4 — original WB supplementary figure 5A [file 41419_2022_5368_MOESM4_ESM.jpg]

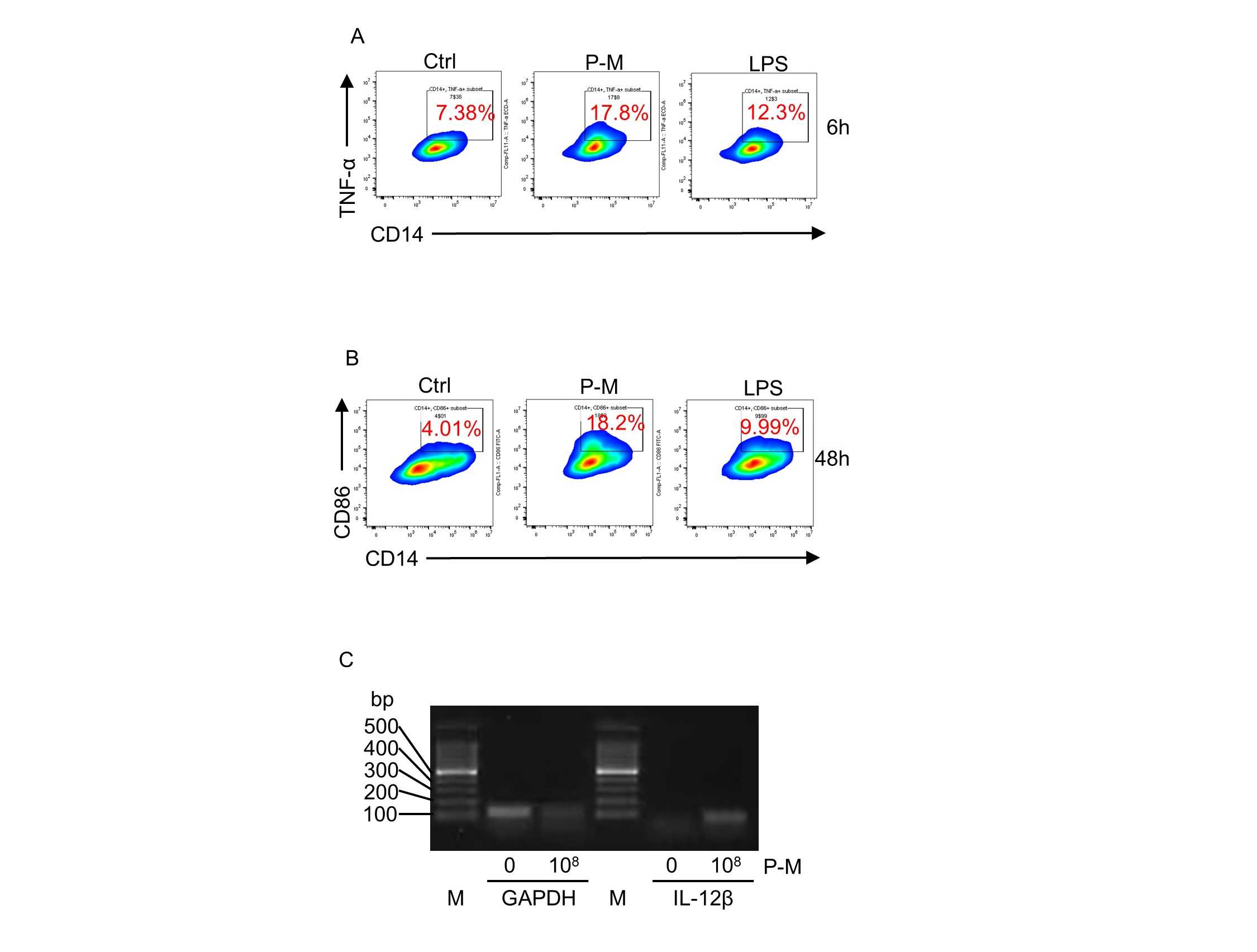

Supplement: Supplementary file 7 — Supplementary Figure 1 [file 41419_2022_5368_MOESM7_ESM.jpg]

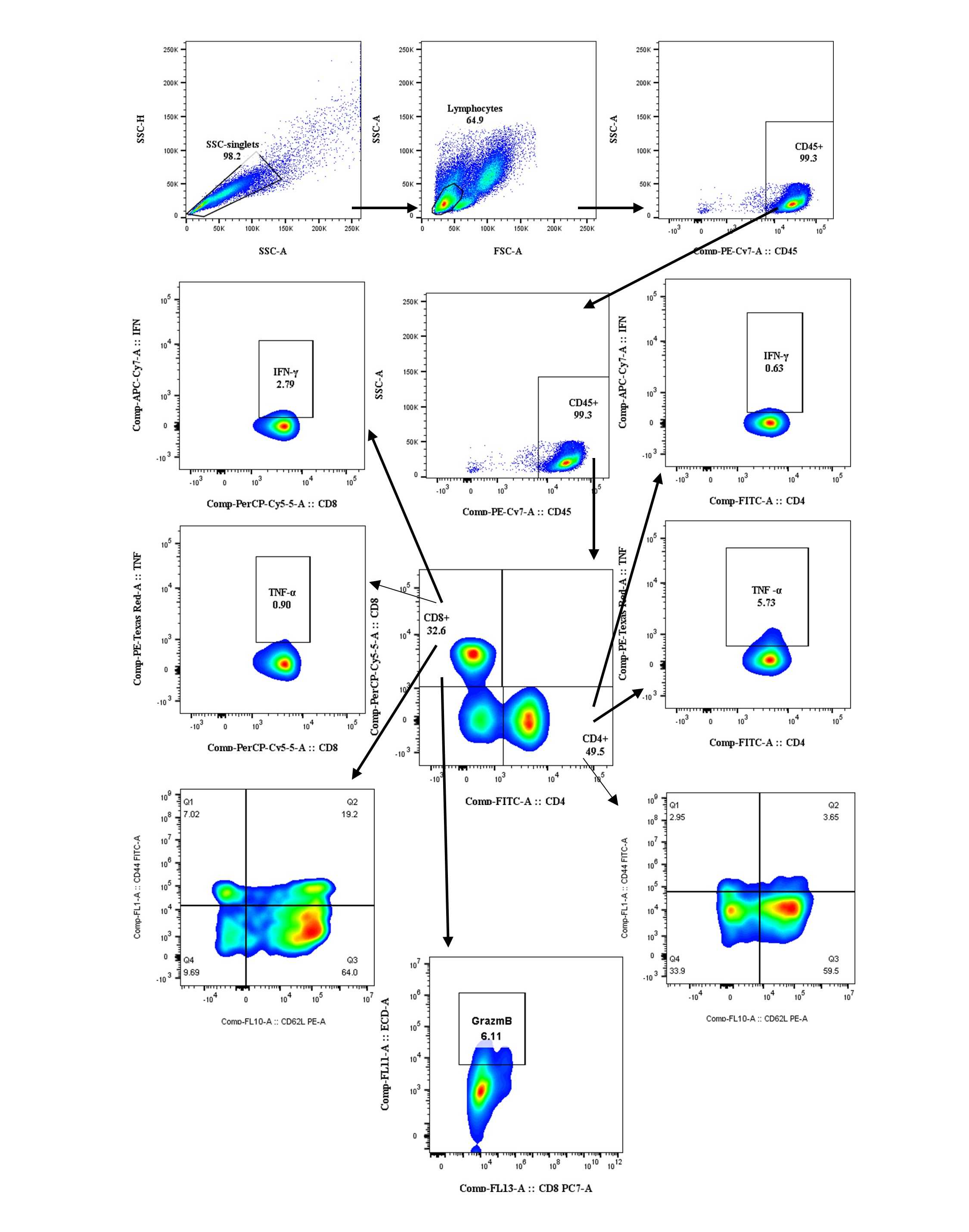

Supplement: Supplementary file 8 — Supplementary Figure 2 [file 41419_2022_5368_MOESM8_ESM.jpg]

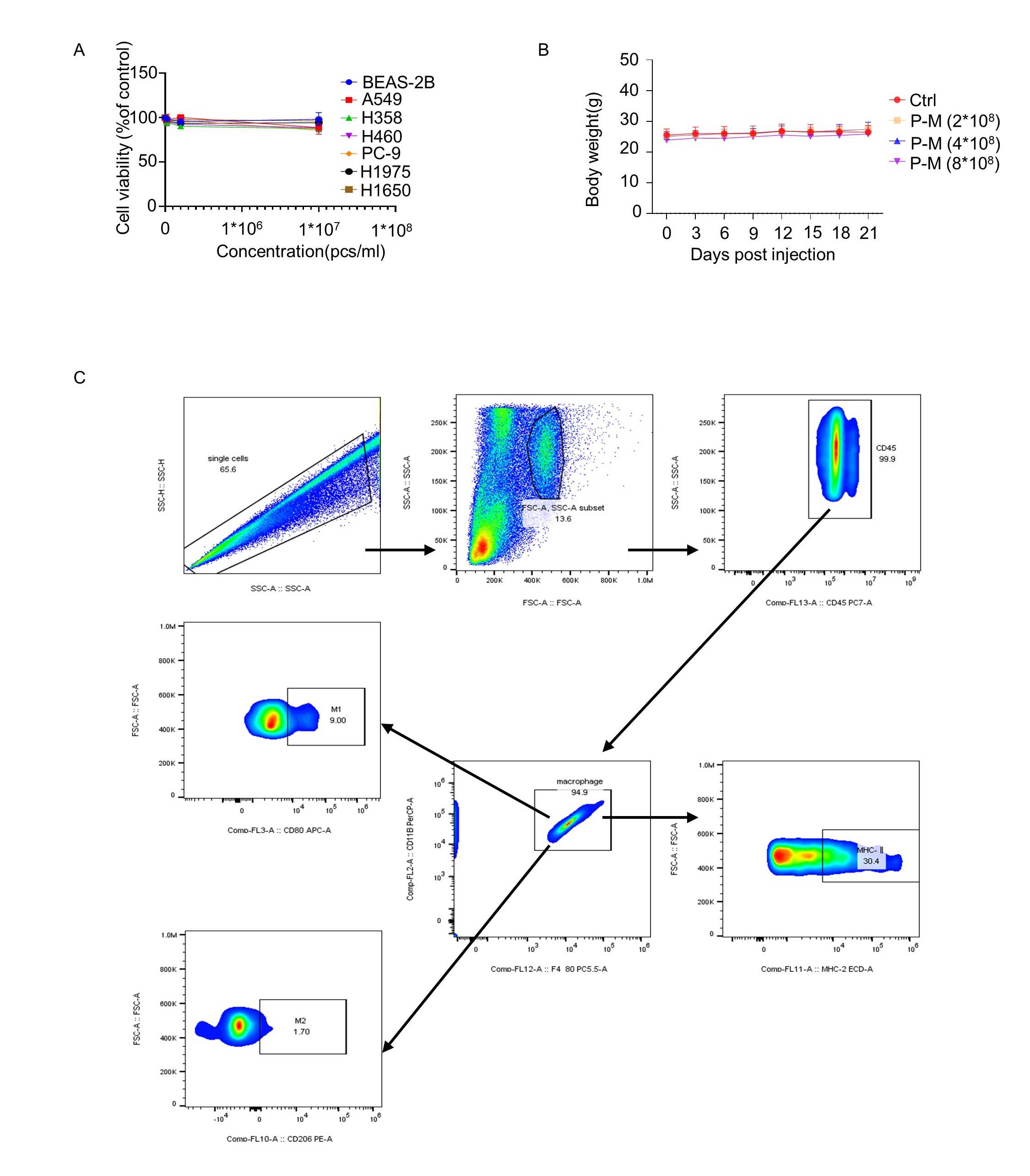

Supplement: Supplementary file 9 — Supplementary Figure 3 [file 41419_2022_5368_MOESM9_ESM.jpg]

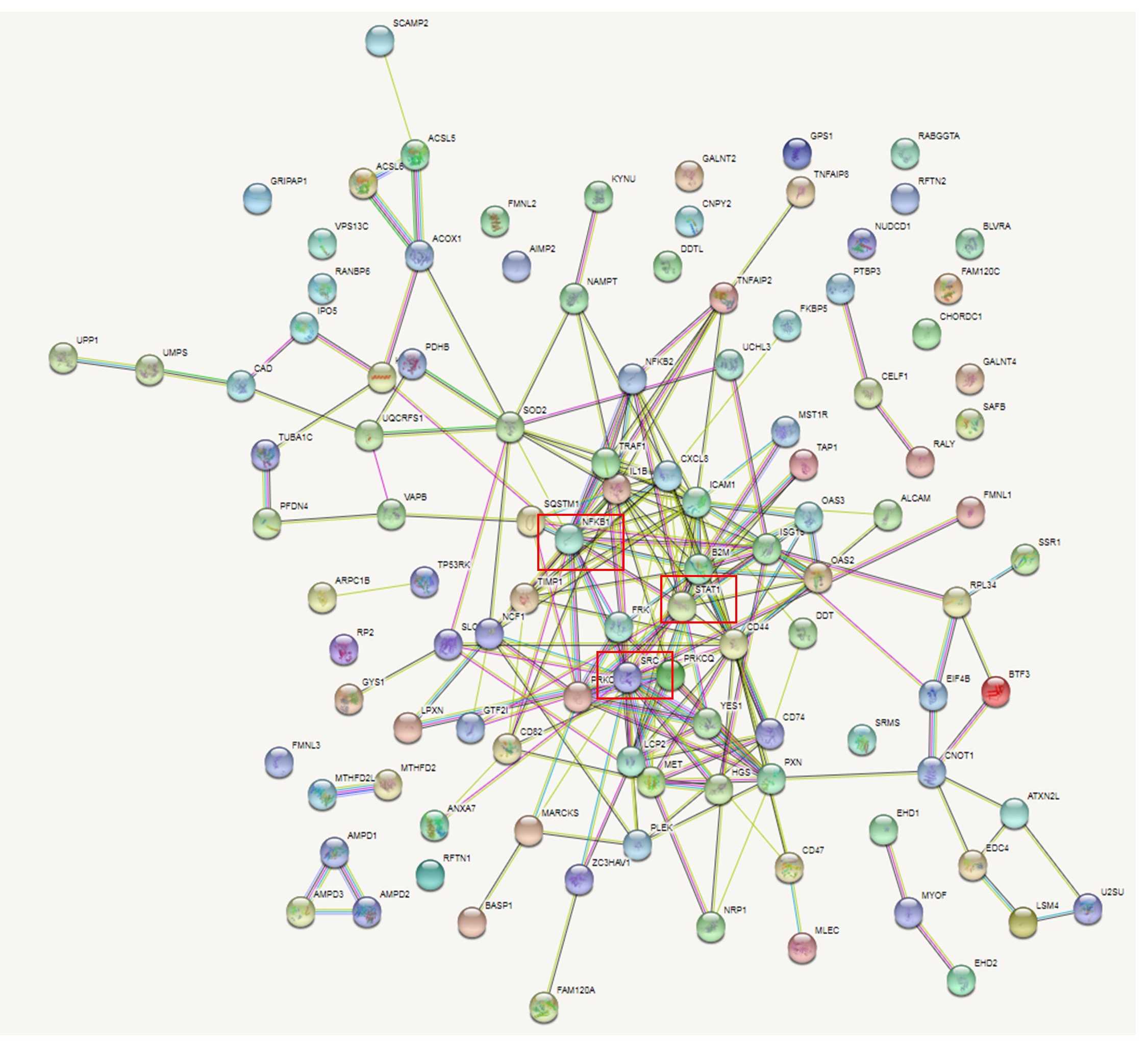

Supplement: Supplementary file 10 — Supplementary Figure 4 [file 41419_2022_5368_MOESM10_ESM.jpg]

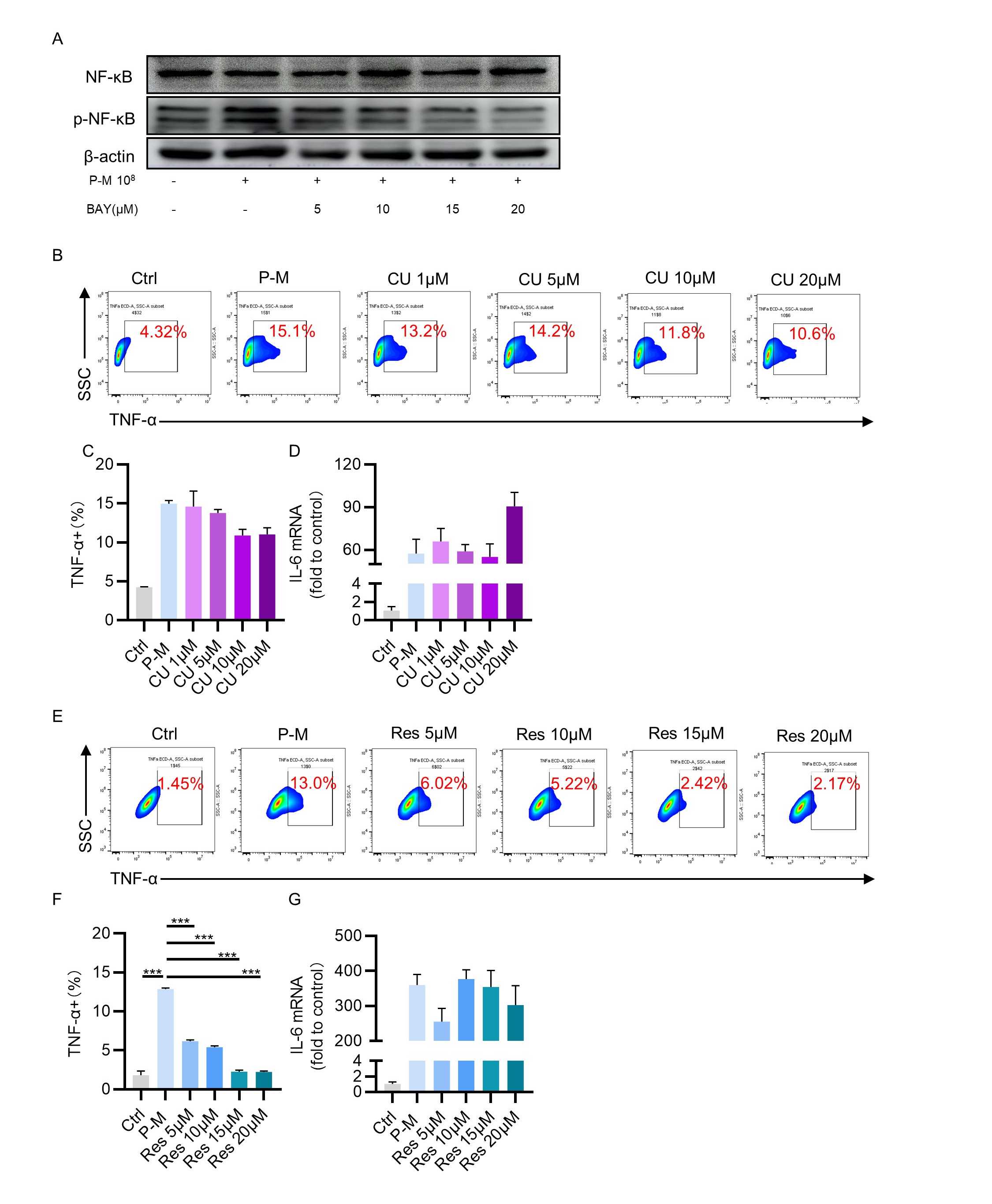

Supplement: Supplementary file 11 — Supplementary Figure 5 [file 41419_2022_5368_MOESM11_ESM.jpg]

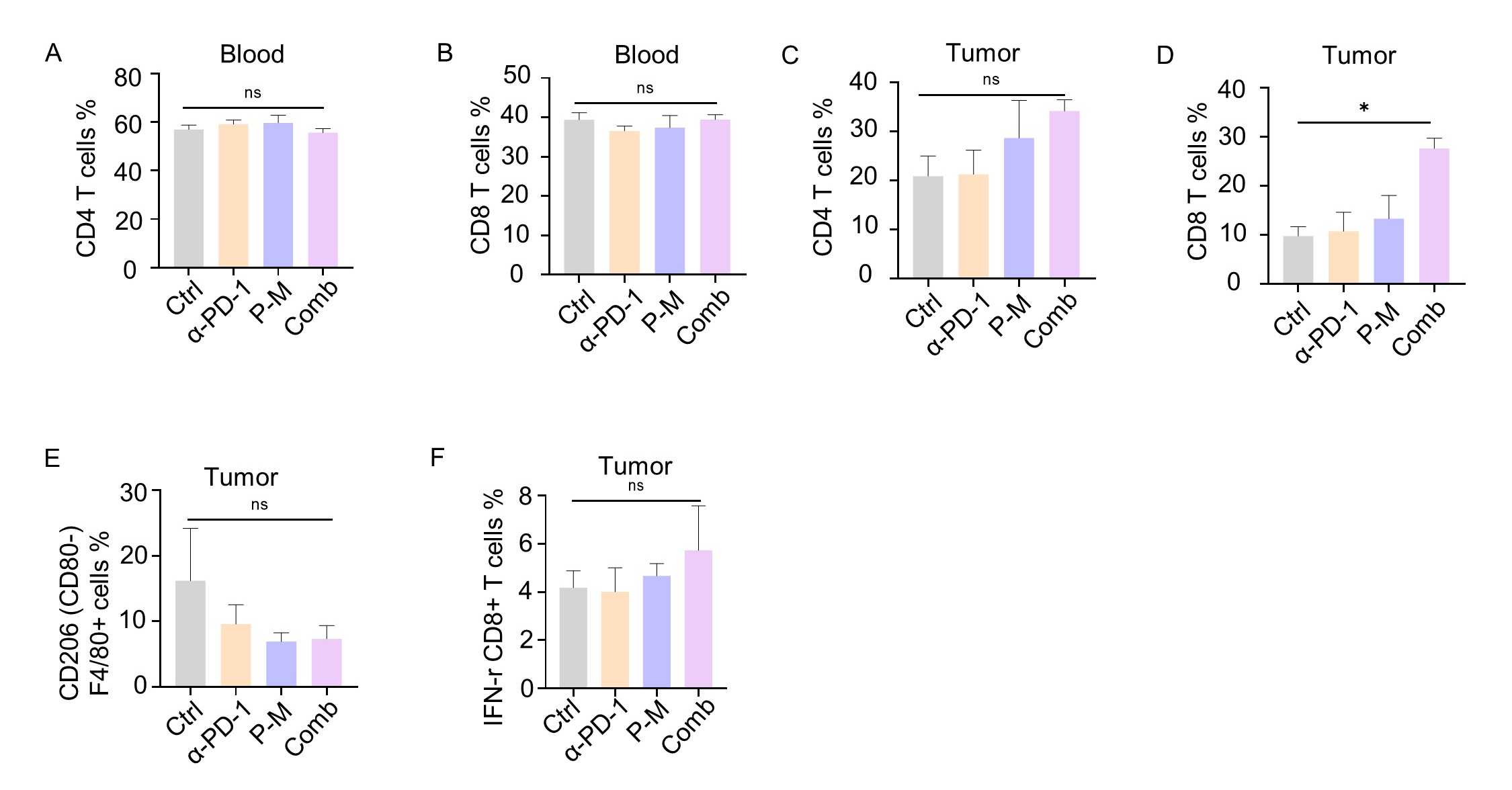

Supplement: Supplementary file 12 — Supplementary Figure 6 [file 41419_2022_5368_MOESM12_ESM.jpg]
