# Supplementary material for: PA-MSHA induces inflamed tumor microenvironment and sensitizes tumor to anti-PD-1 therapy
Source: Cell Death Dis. 2022 Nov 7;13(11):931. doi: 10.1038/s41419-022-05368-6 (PMC9640707; doi:10.1038/s41419-022-05368-6)
Supplement: Supplementary file 3 — supplementary legends [file 41419_2022_5368_MOESM3_ESM.docx]

**Figure S1.**

(A) The expression of TNF-α at 6 hours and (B) M1 marker CD86 at 48 hours in THP-1-derived macrophages was detected by FACS after PA-MSHA treatment. LPS (100μg) was used as positive control. (C) The mRNA level of IL-12β detected by agarose gel electrophoresis of RT-qPCR products. P-M is short for PA-MSHA.

**Figure S2.**

Flow cytometry gating strategy used for the T cells experiments.

**Figure S3.**

(A) NSCLC cells were treated with PA-MSHA, and cell viability was measured by MTT assays. (B) Body weight of the conventional LLC-bearing mice (n=6). (C) Gating strategy for the macrophage cells experiments. P-M is short for PA-MSHA. (*P < 0.05, **P < 0.01, ***P＜0.001, ****P＜0.0001.)

**Figure S4.**

Proteomics and clustering analysis screened out the regulated proteins by PA-MSHA in THP-1 derived macrophages. The results of clustering analysis showed that NF-κB, STAT1, and SRC were identified as three mostly correlated targets.

**Figure S5.**

(A) NF-κB inhibitor: BAY 11-7082 effectively suppressed the phosphorylation of NF-κB induced by PA-MSHA. (B-D) Treatment with TLR8 inhibitor (CU-CPT-8m) didn’t suppress the enhancement of TNF-α and IL-6 in PA-MSHA treated cells. (E-G) TLR4 inhibitor (Resatorvid) successfully blocked the expression of TNF-α but failed to inhibit the increase of IL-6 induced by PA-MSHA. P-M is short for PA-MSHA. (*P < 0.05, **P < 0.01, ***P＜0.001, ****P＜0.0001.)

**Figure S6.**

(A-B) The expression of CD4 and CD8 T cells in blood were not significantly statistic. (C-D) Percentage of CD4 T cells and CD8 T cells in tumor were all up-regulated after combined treatments. (E) Percentage of M2 (CD80^-^) macrophages were downregulated in combined group. (F)Percentage of IFN-γ of CD8^+^ T cells in tumor were up-regulated after combined treatments. P-M is short for PA-MSHA. (*P < 0.05, **P < 0.01, ***P＜0.001, ****P＜0.0001.)
