# Supplementary material for: PA-MSHA induces inflamed tumor microenvironment and sensitizes tumor to anti-PD-1 therapy
Source: Cell Death Dis. 2022 Nov 7;13(11):931. doi: 10.1038/s41419-022-05368-6 (PMC9640707; doi:10.1038/s41419-022-05368-6)
Supplement: Supplementary file 5 — Authors’ contributions [file 41419_2022_5368_MOESM5_ESM.docx]

**Authors’ contributions**

XXF designed this study. MH, FH, YJX, ZBJ, JMH, XPZ, AAN performed experiments. MH, FH, XPZ analyzed the data. MH, FH wrote the manuscript. XXF, DL, JHC, JCH, XMF, ELHL revised the manuscript. XXF reviewed and supervised the experiments. All authors have read and approved the final manuscript.
